# Complete Genome Sequence of Gordonia rubripertincta Bacteriophage Hexbug Suggests Potential for a New CT Subcluster

**DOI:** 10.1128/mra.00773-22

**Published:** 2022-11-02

**Authors:** Daniel J. Barszczak, Maxwell J. Allison, Yaqi Cai, Lily Forman, Joshua M. Goldstein, Sarrah M. Hakimjee, Matthew T. Scapicchio, Ruben Torres, Hannah E. Gavin

**Affiliations:** a Tufts University, Medford, Massachusetts, USA; b Experimental College, Tufts University, Medford, Massachusetts, USA; c Department of Biology, Tufts University, Medford, Massachusetts, USA; DOE Joint Genome Institute

## Abstract

Through the SEA-PHAGES program at Tufts University, a bacteriophage infecting Gordonia rubripertincta NRRL B-16540 was isolated and characterized. Hexbug is a lytic phage and is currently one of 44 phages belonging to cluster CT. The Hexbug genome shares >96% nucleotide identity with cluster CT phage Orla.

## ANNOUNCEMENT

Bacteriophages (phages), which number approximately 10^31^ particles on Earth, are viruses that infect bacterial hosts (1, 2). Since the discovery of these entities in 1915, phages have shown potential to be used to treat problematic bacterial infections (3, 4). In collaboration with the SEA-PHAGES program, here we report the comprehensive genome sequence and annotation of a novel Gordonia rubripertincta phage, Hexbug (5, 6).

**FIG 1:**
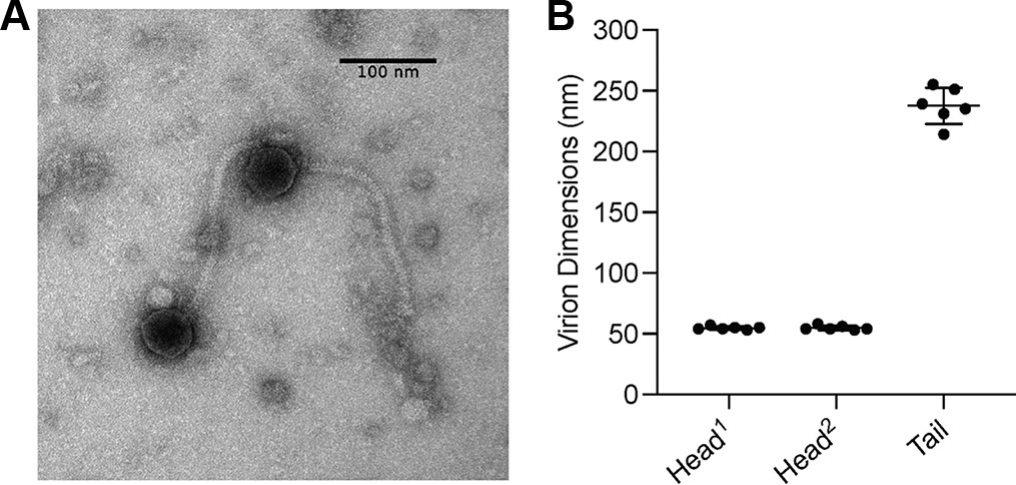
(A) Hexbug virions negatively stained with 1% uranyl acetate and visualized with transmission electron microscopy (TEM) at the Harvard Medical School Electron Microscopy Facility. Hexbug is a siphovirus with an isometric icosahedral head and long noncontractile tail (26). (B) Dimensions of 6 virions measured using ImageJ (27). Graph displays individual measurements as dots, with bars for mean and standard deviation. Scale bar, 100 nm. Hexbug tails measure 238 nm ± 15 nm in length. Capsids are 55 nm ± 1 nm along the axis perpendicular to the tail (Head^1^) and 55 nm ± 2 nm along the same axis as the tail (Head^2^).

Hexbug was isolated from a moist garden soil sample collected in early September 2021 from Arlington, Massachusetts (42.412895 N, 71.13305 W). Briefly, the soil sample was washed with peptone-yeast extract-calcium (PYCa) medium, the wash was collected by centrifugation and filtration (0.02-μm pore size), the filtrate was plated in top agar with Gordonia rubripertincta NRRL B-16540, and the plates were incubated at 30°C. Hexbug was purified by three successive rounds of single-plaque isolation. Negative-stain transmission electron microscopy revealed that Hexbug has siphovirus morphology (Fig. 1).

A high-titer lysate was generated from confluent plates. DNA was then extracted from the lysate with the Promega Wizard DNA clean-up system and prepared for sequencing with the NEBNext Ultra II fragmentation system (FS) prep kit. The library was sequenced on an Illumina MiSeq (v3 reagents) instrument at the University of Pittsburgh, yielding 48,574 150-bp reads with approximately 1,038× coverage (7). The genome sequence was assembled *de novo* from raw reads with Newbler v2.9 and results checked with Consed v29 (8, 9). The Hexbug genome is 47,190 bp long, with a GC content of 63.3% and 10-bp 3′ single-stranded overhangs (5′-CGGTAGGCAT-3′).

Open reading frames (ORFs) in the Hexbug genome were initially autoannotated using GeneMark v4.9, Glimmer v3.02b, and ARAGORN v1.1 within DNA Master v5.0.2 (10–13). Gene starts were then refined using BLASTn searches against the PhagesDB Actinobacteriophage database and NCBI BLASTn standard database nucleotide collection. Information on coding potential, start site prevalence, and Shine Dalgarno sequence potential were gathered from GeneMark, Starterator, and DNAMaster, respectively (10, 13–16). Gene functions were predicted using Phamerator, NCBI BLASTp against standard databases, and HHPred against the following databases: PDB_mmCIF70_12_Oct, Pfam-A_v35, NCBI_Conserved_Domains(CD)_v3.18, and PRD_v6.9 (14, 17, 18). The genome was screened for antibiotic resistance genes on the CARD v3.2.0 RGI v5.2.1 Web portal (19). ORFs that could not be assigned a function were evaluated for transmembrane potential with TMHMM v2.0 and SOSUI (20–22).

Annotation identified 73 open reading frames, of which 36 were assigned functions. Genes connected to virion structure and assembly (*n* = 16) and genes facilitating cell lysis (*n* = 3) are situated in the left half of the genome. ORFs connected to DNA metabolism, including helicase and primase genes, are distributed across the right side of the genome. Five additional ORFs possess predicted transmembrane domains.

Actinobacteriophages that share at least 35% gene content similarity (GCS) are grouped into clusters; members often infect related hosts and show similar viral replication modes (4, 7, 23, 24). Hexbug is assigned to cluster CT according to the GCS analysis reflected in the Actinobacteriophage database as of 31 May 2022. Like other CT phages, Hexbug produces clear plaques and lacks lysogeny-associated genes, suggesting an obligately lytic replication cycle (7). The isometric capsid shape of Hexbug (Fig. 1) is also common in cluster CT.

Within cluster CT, Hexbug is most similar to phage Orla (GenBank accession MN89453), sharing 97% nucleotide identity across 99% of the genome. At >93%, the GCS between Hexbug and Orla is greater than their relationship to any other cluster CT phages and is comparable to GCS within phage subclusters (23). The relationship between these two phages foreshadows the emergence of the first phage subcluster within cluster CT. In application, lytic phages like Hexbug expand the arsenal of putative therapeutics for rare Gordonia rubripertincta infections in humans (25).

### Data availability.

The complete Hexbug genome is available at GenBank accession number ON970609. The raw data can be accessed using Sequence Read Archive (SRA) number SRX14443511.
